# Immunogenicity and safety of co-administered *Escherichia coli*-produced bivalent HPV-16/18 vaccine and hepatitis E vaccine

**DOI:** 10.3389/fimmu.2026.1837539

**Published:** 2026-05-29

**Authors:** Rui Yan, Mengting Cen, Yang Zhou, Liyang Ma, Xiaofei Zhou, Hanqing He, Xiaohua Qi, Xuewen Tang, Hui Liang, Yaping Chen, Jiaxing Qi, Shigui Yang

**Affiliations:** 1 Zhejiang Provincial Center for Disease Control and Prevention, Zhejiang Key Lab of Vaccine, Infectious Disease Prevention and Control, Hangzhou, Zhejiang, China; 2 Department of Epidemiology and Biostatistics, School of Public Health, Department of Emergency Medicine, Second Affiliated Hospital, Zhejiang University School of Medicine, Hangzhou, Zhejiang, China

**Keywords:** bivalent, co-administration, *Escherichia coli*, HEV vaccine, human papillomavirus vaccine, immunogenicity, non-inferiority

## Abstract

**Background:**

Human papillomavirus (HPV) and Hepatitis E virus (HEV) infections are major health concerns for women of reproductive age. Administration of HPV and HEV vaccines serves as primary prevention strategies. Our study aimed to demonstrate the immunogenicity and safety of co-administering *Escherichia coli*-produced bivalent HPV-16/18 vaccine and HEV vaccine compared to HPV-alone/HEV-alone.

**Methods:**

This randomized, multicenter, open-label trial was conducted at 4 clinical study sites in Zhejiang Province, China. Participants were enrolled and randomly assigned into three groups to receive bivalent HPV vaccine co-administered with HEV vaccine, HPV vaccine alone and HEV vaccine alone following a 0, 1, 6-month vaccination schedule. The geometric mean concentrations (GMC) and seroconversion rates were measured at months 0 and 7 to evaluate immunogenicity. Non-inferiority was met if the lower bound of the two-sided 95% CI for the seroconversion rate difference exceeded -5%, or that for the GMC ratio exceeded 0.5. Safety outcomes were assessed post-vaccination.

**Results:**

Of the 480 women aged 18–25 years enrolled, 462 (96.3%) completed the full vaccination regimen and follow-up and were included in the per-protocol set (PPS), whereas all 480 participants (100%) were included in the safety set (SS). The GMC ratio for HPV-16 (HPV+HEV group vs. HPV-alone group) was 0.82 (95% CI 0.68–0.98), with corresponding GMCs of 433.16 IU/mL and 529.20 IU/mL, respectively. For HPV-18, the GMC ratio (HPV+HEV vs. HPV-alone) was 0.78 (95% CI 0.64-0.96), based on concentrations of 318.30 and 407.54 IU/mL, respectively. HEV GMCs were 10.51 WU/mL for the co-administration group and 11.18 WU/mL for the HEV-alone group (ratio 0.94; 95% CI 0.81-1.09). All groups achieved 100% seroconversion. Most adverse reactions were Grade 1, and no vaccine-related serious adverse events (SAEs) occurred.

**Conclusion:**

Co-administration of HPV vaccine and HEV vaccine elicited high seroconversion rates and met non-inferiority criteria compared to administration of each vaccine alone, with a similar safety profile, supporting the feasibility of concomitant vaccination.

## 1 Introduction

Human papillomavirus (HPV) is a highly prevalent sexually transmitted infection, with certain high-risk types, particularly HPV-16 and HPV-18, being the primary causative agents of cervical cancer, the fourth most common cancer in women globally (1). Persistent infection with these oncogenic strains is responsible for over 70% of cervical cancer cases worldwide, as well as a significant proportion of other anogenital cancers (including vaginal, vulvar, anal, and penile cancers) and oropharyngeal cancers (2, 3). Therefore, effective preventive measures are necessary and important. The WHO and the Advisory Committee on Immunization Practices (ACIP) recommend individuals aged 9 through 45 years to receive HPV vaccination, particularly adolescents and young adults before initiating sexual activity (4, 5). An *Escherichia coli*-produced HPV 16 and 18 bivalent vaccine named Cecolin was launched in China on December 31, 2019 (6), with a two-dose schedule (at months 0 and 6) for girls aged 9–14 years and a three-dose schedule (at months 0, 1, and 6) for women aged 15–45 years. Clinical trial results (7, 8) demonstrate that the vaccine can effectively induce high levels of antibodies, achieve 100% efficacy against HPV-16/18 related precancerous lesions and maintain long-term efficacy. It also exhibits a favorable safety profile.

Hepatitis E is an emerging health killer caused by infection of hepatitis E virus (HEV), which belongs to the family Herpesviridae and primarily transmitted via fecal-oral route. Hepatitis E infection is widespread globally with a higher prevalence in low- and middle-income countries. HEV infection imposes a heavy public health burden worldwide. WHO reported that HEV infection accounted for 5.4% of global disability-adjusted life years (DALYs) related to acute hepatitis in 2021 (9). HEV infection poses a threat to diverse demographic groups, particularly women in child-bearing age. Studies have found that pregnant women are particularly susceptible to HEV infection (10–12). They face an increased risk of developing fulminant hepatic failure. Additionally, the virus can be transmitted vertically to the fetus, elevating the likelihood of miscarriage, premature birth, and stillbirth. Vaccination against HEV has become an effective measure to prevent HEV infection and control the progression of hepatitis E. Currently there is only one hepatitis E vaccine approved for use worldwide. Hecolin^®^, the recombinant HEV vaccine licensed in China in 2011, has been demonstrated to exhibit favorable immunogenicity and a good safety profile, and was subsequently approved for use in Pakistan (13). A large-scale phase III clinical trial revealed that, following the standard 3-dose vaccination schedule (administered at 0, 1, and 6 months), the hepatitis E vaccine maintains an efficacy of 86.6% for up to 10 years (14).

Implementing co-administration of the HPV vaccine with other routine vaccinations is an effective measure to reduce immunization costs and improve vaccination compliance. The HPV vaccine has been demonstrated to be co-administered with routine vaccines—such as hepatitis B vaccine (15), hepatitis A and B vaccine (16), REPEVAX (diphtheria, tetanus, acellular pertussis and inactivated poliomyelitis vaccine) (17), MCV4+ Tdap (18), and MenACWY-CRM (19)—without compromising immunogenicity or safety. The hepatitis E vaccine, when co-administered with the hepatitis B vaccine, also demonstrates favorable immunogenicity and safety (20). The identical standard immunization schedules of the HEV and HPV vaccines create a perfect opportunity for their simultaneous administration. No previous studies have discussed the co-administration of the HPV and HEV vaccines. This paper will preliminarily investigate the immunogenicity and safety of simultaneously administering the bivalent HPV-16/18 vaccine (*Escherichia coli*-produced) and the HEV vaccine, providing a new perspective and potential for the synergistic prevention of HPV and HEV.

## 2 Materials and methods

### 2.1 Study design and participants

This study was a randomized, multicenter, open-label trial conducted in Zhejiang Province between October 2021 and Dec 2023. It was approved by the Ethics Review Committee of the Zhejiang Provincial Center for Disease Control and Prevention (approval number: 2021-021-01; approval date: 26 July 2021). Participants were recruited at four clinical study sites in Zhejiang Province: Wengrong Hospital (Hengdian, Dongyang), Dongshan Community Health Service Center (Jiaojiang, Taizhou), Linhai Maternal and Child Health Hospital, Taizhou Hospital. We restricted the age of the participants to be over 18 years old in this study. Healthy females were enrolled if they met the following inclusion criteria: aged 18–25 years; axillary temperature lower than 37.0 °C; negative urine pregnancy test; and willingness to comply with the procedure of the study. Participants were excluded if they had any preexisting severe, acute or chronic disease, history of severe anaphylaxis with vaccines or history of any HPV or HEV vaccination. Detailed inclusion and exclusion criteria can be found in Appendix 1 of the **Supplementary Materials**.

### 2.2 Randomization and masking

**Figure 1:**
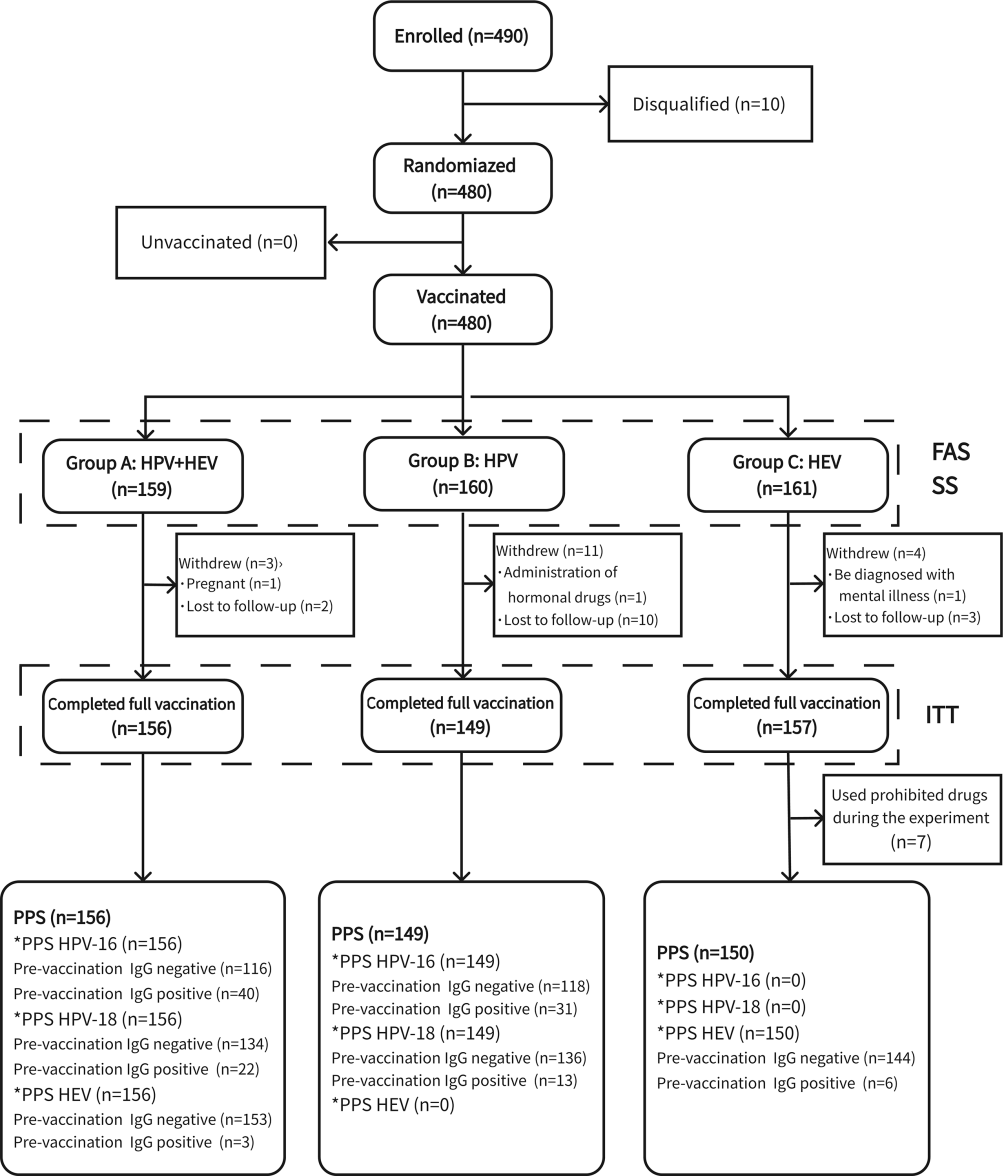
Participant recruitment flow diagram. FAS, Full Analysis Set. This cohort included all randomized subjects who received at least one dose, regardless of whether they received the second dose. ITT, Intention To Treat Set. This cohort included subjects who received at least one dose of the vaccine and completed valid serological visits at months 0 and 7. PPS, per-protocol set for immunogenicity. This cohort consisted of women who received all three doses of vaccines, obtained corresponding antibody data for 0-m and completed 7-m serum samples during the protocol-specified time frames, and committed no important violations of the protocol. SS, safety analysis set. All participants at enrollment were included in the SS dataset.

Eligible women were randomized (1:1:1) according to computer-generated allocation schedules to receive either three doses of the bivalent HPV-16/18 vaccine co-administered with the HEV vaccine (Group A), the bivalent HPV-16/18 vaccine alone (Group B), or the HEV vaccine alone (Group C), based on the group assignments in the sequentially opened sealed opaque randomization envelopes (**Figure 1**). The corresponding randomization numbers were listed to form a randomization code list, and sequentially numbered, opaque, sealed envelopes were prepared accordingly. Investigators assigned the randomization numbers in ascending order according to the participant enrollment sequence, opened the sealed envelopes one by one, and each participant received the vaccine as indicated by the envelope. Researchers involved in different stages were independent of each other and had no knowledge of the allocation sequence in the other stages, ensuring allocation concealment and blinding. The laboratory personnel measuring the immunogenicity results were unaware of the treatment allocation. This study was conducted in accordance with the Declaration of Helsinki and Good Clinical Practice guidelines. All participants provided written informed consent, and serum samples were anonymized and encrypted throughout the study.

### 2.3 Vaccines and procedures

The bivalent HPV vaccine used in the trial was a mixture of two aluminum hydroxide adjuvant-absorbed recombinant L1 virus-like particles (VLPs) of HPV-16 and HPV-18, expressed in Escherichia coli (Cecolin, Xiamen Innovax, Xiamen, China; Batch No. B202105058). The formulation contained 40 μg of HPV-16 L1 VLP and 20 μg of HPV-18 L1 VLP, suspended in 0.5 mL of buffered saline with 208 μg of aluminum adjuvant. The HEV vaccine used in the trial was developed by Xiamen Innovax Biotech Co., Ltd., China (Batch No. A202101001) and contained 30 μg of recombinant HEV capsid antigen adsorbed onto 277 μg of aluminum adjuvant, suspended in 0.5 mL of buffered solution.

The vaccine was administered by intramuscular injection at the attachment site of the deltoid muscle on the outer side of the upper arm. All participants received the vaccine at day 0, month 1 (28–60 d), and month 6 (150–240 d) according to their assigned group. For participants in group A, HPV vaccine and HEV vaccine injections were co-administered at separate anatomic sites (i.e., one in the left arm and one in the right). For participants in Groups B and C, the vaccine was administered in the left arm at Month 0, the right arm at Month 1, and the left arm again at Month 6. Serum samples were collected at baseline (before the first vaccination) and 30–60 days after the third vaccination for all participants.

### 2.4 Statistical analysis

#### 2.4.1 Sample size

The sample size was calculated using PASS software (version 11.0), mainly based on the assumption that simultaneous vaccination elicits non-inferior immunogenic responses in terms of high seroconversion rates compared to monovalent vaccination. We assumed HPV16-IgG and HPV18-IgG seroconversion rates of 99.0% in Groups A and B, and a 99.5% HEV-IgG seroconversion rate in Groups A and C. These assumptions were based on previous studies showing 100.0% seroconversion rates6 for HPV16-IgG and HPV18-IgG one month after completion of the three-dose bivalent HPV-16/18 vaccine (E. coli-produced), and a 99.86% (21) HEV-IgG seroconversion rate one month after the third dose among baseline seronegative individuals. Calculation processes are detailed in Appendix 2 of the **Supplementary Materials**.

According to FDA guidance (22), the non−inferiority margin is determined based on the acceptable loss of clinical efficacy, and a 50% retention of the reference vaccine’s effect is commonly used as the criterion for non−inferiority (23, 24). Therefore, we chose 0.5 as the cut−off for the geometric mean concentration (GMC) ratio. For the seroconversion rate difference, we referred to previous studies (14, 25, 26) and defined non−inferiority as the lower bound of the 95% confidence interval ≥ –5%. Using a one-sided significance level of 0.025, sample sizes for Groups A and B (for HPV) and Groups A and C (for HEV) were calculated at 80% power. The required sample size per group was 139, which, after adjusting for a 10% baseline seropositivity rate and a 5% dropout rate, increased to approximately 160 participants per group. Thus, the Co-administration HPV and HEV group (Group A), HPV vaccine group (Group B), and HEV vaccine group (Group C) will each enroll about 160 participants, totaling approximately 480.

A post−hoc power analysis using the observed GMC variability confirmed that the achieved sample size provides >99% power for the GMC ratio non−inferiority endpoint (non−inferiority margin 0.5; see **Supplementary Appendix 3**).

#### 2.4.2 Immunogenicity assessment

The primary outcome of this study was to assess the non-inferiority of the co-administration group versus the single vaccine groups by comparing the GMC of antibodies and the seroconversion rate between different groups of the protocol set. Immunogenicity was primarily analyzed in the per-protocol set (PPS), which included all participants who had complied with the protocol and had available IgG antibody results at Month 7. A sensitivity analysis was also performed in the intention−to−treat (ITT) set, comprising all participants who completed the month-7 serology visit regardless of protocol adherence. IgG antibodies against HPV-16 and HPV-18 were measured at baseline (day 0) and Month 7 by enzyme-linked immunosorbent assay (ELISA) (27). Positive samples were quantified using reference materials traceable to the WHO international standards for anti–HPV-16 (NIBSC code 05/134) and anti–HPV-18 (NIBSC code 10/140), and results were expressed in international units per milliliter (IU/mL). The lower limits of detection (LLOD) were 3.0 IU/mL for HPV-16 antibodies and 2.1 IU/mL for HPV-18 antibodies. Anti-HEV IgG was tested qualitatively and quantitatively by ELISA according to the manufacturer’s instructions, as previously described (26). Positive samples were further quantified and expressed in WHO units per milliliter (WU/mL); the lower limit of quantification (LLOQ) was 0.03 WU/mL. For GMC calculations, antibody titers below the LLOD were assigned half the cutoff value. Seroconversion was defined as (1) a change from below to at or above the LLOD/LLOQ (HPV-16: 3.0 IU/mL; HPV-18: 2.1 IU/mL; HEV: 0.03 WU/mL) after vaccination, or (2) a ≥4-fold rise in antibody titer when the baseline concentration was already at or above the respective LLOD/LLOQ.

#### 2.4.3 Safety assessment

Secondary outcomes included adverse events and adverse reactions after vaccination. After each dose, all participants remained at the vaccination site for 30 minutes of observation for immediate adverse reactions (ARs). They were required to record all adverse events (AEs) occurring within 30 days after each vaccination in diary cards accurately and properly. All serious adverse events (SAEs) and serious adverse reactions (SARs) were recorded throughout the trial. AEs and ARs of each system were classified by severity into grade 1, 2 and 3. Definition of solicited and unsolicited adverse events: Events occurring within 7 days of vaccination were considered solicited adverse events; Events occurring after 7 days but within 30 days were classified as unsolicited adverse events; Non-key events occurring within 30 days of vaccination were also classified as unsolicited events. The number and incidence of solicited and unsolicited adverse events and adverse reactions were summarized according to System Organ Class (SOC) and Preferred Term (PT).

#### 2.4.4 Statistical analysis

Immunogenicity was analyzed in PPS dataset, and safety was assessed in SS dataset. Seropositive rates and GMCs were summarized with 95% confidence intervals (CIs), calculated using the Clopper–Pearson method and the Student’s t-distribution, respectively. After the whole immunization schedule, if the lower limit of the 95% confidence interval for the difference in seroconversion rates between groups exceeds -5%, or the lower limit of the 95% CI for the GMC ratio is greater than 0.5, the co-administration group will be considered non-inferior to the groups receiving only the HPV-16/18 vaccine or only the HEV vaccine. Statistical analyses were performed by an independent statistician using R software (version 4.4.2). All statistical tests were two-sided, and a P value < 0.05 was considered statistically significant.

## 3 Results

A total of 480 females were enrolled and randomized into 3 groups (group A: HPV+HEV, n=159; group B: HPV, n=160; group C: HEV, n=161). 480 participants were all included in SS dataset. 18 participants (3.75%) dropped out of the trial but remained in SS dataset (**Figure 1**). 462 participants composed PPS dataset for immunogenicity, including 156 participants in group A, 149 participants in group B, 157 participants in group C that finished 3 doses of vaccination and had a valid post-vaccination serology result within the protocol specified time. The baseline demographic characteristics of each group are summarized in **Table 1**. Overall, the mean age was 19.56 ± 2.02 years. All participants exhibited axillary temperatures within the normal reference range (36.45 ± 0.32 °C). Each of them demonstrated normal cardiopulmonary auscultation and was free of comorbidities at baseline. Demographic and baseline characteristics of the participants were generally well balanced between groups.

**Table 1:** Study population characteristics at baseline.

Characteristics	Group A: HPV+HEV	Group B: HPV	Group C: HEV	Total
N	159	160	161	480
Mean age, years±SD	19.55±2.02	19.56±2.08	19.56±1.97	19.56±2.02
Sex, n (%)				
Female	159(100.0)	160(100.0)	161(100.0)	480(100.0)
Axillary thermometer, °C (SD)	36.44(0.33)	36.44(0.33)	36.48(0.30)	36.45(0.32)
Normal auscultation of the heart and lungs, n (%)				
Yes	159(100.0)	160(100.0)	161(100.0)	480(100.0)
No	0(0.00)	0(0.00)	0(0.00)	0(0.00)
Combined diseases, n (%)				
Yes	0(0.00)	0(0.00)	0(0.00)	0(0.00)
No	159(100.0)	160(100.0)	161(100.0)	480(100.0)
Seronegativity status at baseline, n (%)				
HPV16 IgG	117(73.58)	126(78.75)	–	–
HPV18 IgG	137(86.16)	146(91.25)	–	–
HEV IgG	156(98.11)	–	155(96.27)	–

Prior to vaccination, the majority of participants in the PPS cohort were seronegative for antibodies against HPV-16, HPV-18 and HEV. Baseline serological testing showed that 117 (73.58%) participants in Group A and 126 (78.75%) in Group B were seronegative for HPV-16 IgG. For HPV-18 IgG, 137 (86.16%) participants in Group A and 146 (91.25%) in Group B were seronegative. For HEV IgG, 156 (98.11%) participants in Group A and 155 (96.27%) in Group C were seronegative. There was no statistically significant difference in the pre-vaccination seronegative rate for HPV-16 or HPV-18 IgG between Group A and Group B, or for HEV IgG between Group A and Group C (**Table 1**).

**Figure 2:**
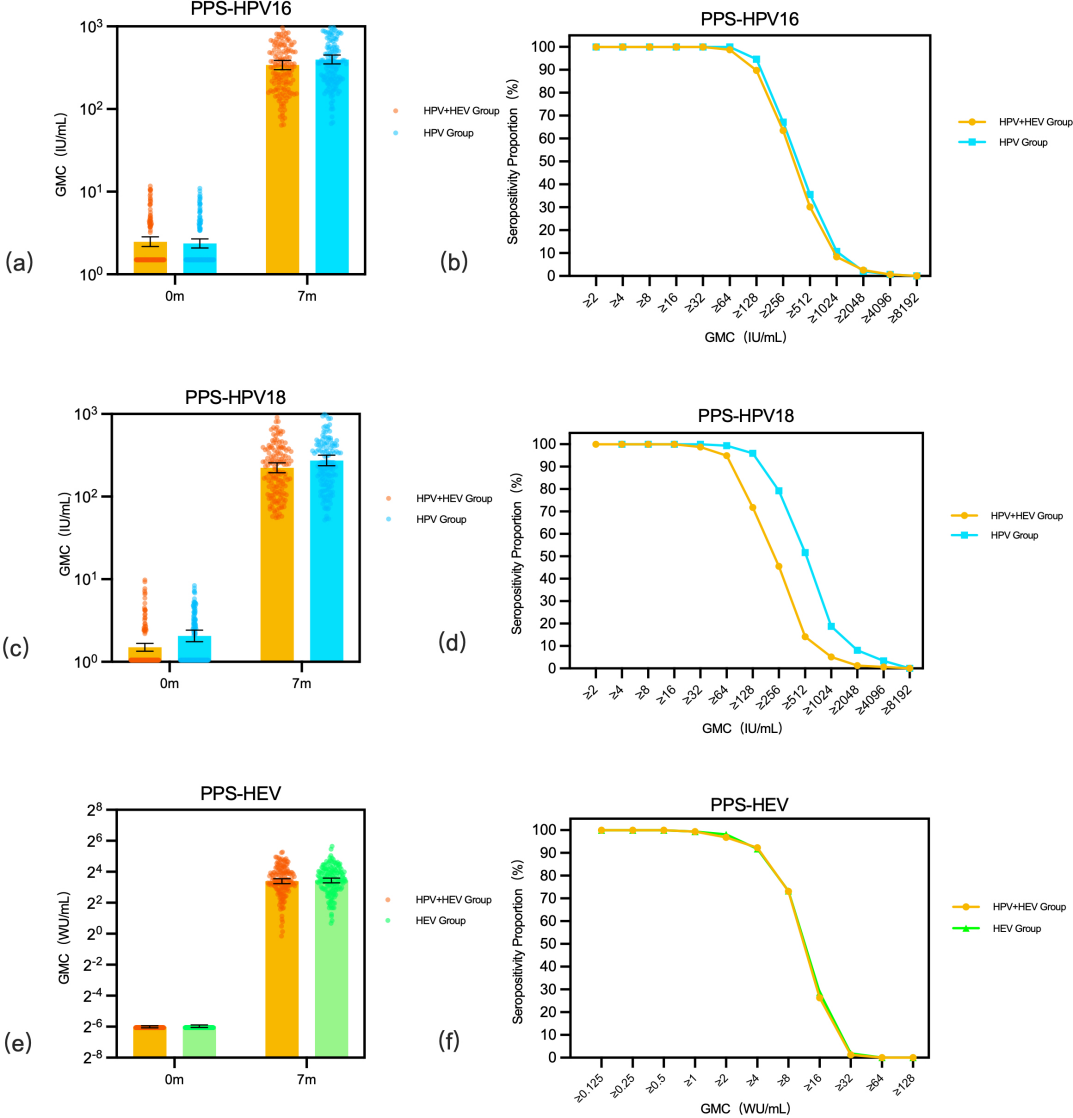
**(a)** The geometric mean concentration of HPV16-IgG antibodies at 0 and 7 months. **(b)** Reverse cumulative curves of HPV16-IgG antibodies at 7 Month. **(c)** The geometric mean concentration of HPV18-IgG antibodies at 0 and 7 months. **(d)** Reverse cumulative curve of HPV18-IgG antibodies at 7 Month. **(e)** The geometric mean concentration of HEV-IgG antibodies at 0 and 7 months. **(f)** Reverse cumulative curve of HEV-IgG antibodies at 7 Month.

After full vaccination, all participants who were seronegative for HPV-16, HPV-18, or HEV IgG at baseline became seropositive, and those who were seropositive showed ≥ 4-fold GMC increases. The 95% CIs for the seroconversion-rate differences (Group A vs. B for HPV16/18-IgG and Group A vs. C for HEV-IgG) were all greater than -5%, confirming non-inferiority of the co-administration group. (**Figure 2**).

Post-vaccination HPV-16 IgG GMCs were 433.16 IU/mL (95% CI: 379.70 IU/mL–494.15 IU/mL) in group A and 529.20 IU/mL (95% CI: 466.89 IU/mL–599.81 IU/mL) in group B, yielding a GMC ratio (A/B) of 0.82 (95% CI: 0.68–0.98). The 95% CI lower bound exceeded the 0.5 non-inferiority margin, confirming non-inferiority for HPV-16. Similarly, HPV-18 GMCs were 318.30 IU/mL (95% CI: 276.64 IU/mL–366.24 IU/mL) in group A and 407.54 IU/mL (95% CI: 350.50 IU/mL–473.86 IU/mL) in group B, with a ratio of 0.78 (95% CI: 0.64–0.96) and a lower bound above 0.5. For HEV, group A and C GMCs were 10.51 WU/mL (95% CI: 9.43 WU/mL–11.71 WU/mL) and 11.18 WU/mL (95% CI: 10.11 WU/mL–12.37 WU/mL), respectively, giving a ratio of 0.94 (95% CI: 0.81–1.09); the lower bound of 0.81 likewise exceeded the 0.5 margin. (**Table 2**) Consistent findings were observed in the sensitivity analysis using the ITT dataset (**Supplementary Appendix 6**), confirming the robustness of the non−inferiority conclusions.

**Table 2:** Seroconversion rates and GMCs at Month 7(PPS cohort).

Groups			N	Seroconversion Rate	GMC at 7m		Non-inferiority analysis	
						95% CI	Rate Difference^a^	GMC ratio^b^
Group A: HPV+HEV	HPV-16(IU/mL)	Total	156	100%	433.16	379.70, 494.15	0.00(-2.40, 2.51)	0.82(0.68, 0.98)
		Positive pre-vaccination	40	100%	452.62	350.76, 584.06	0.00(-8.76, 11.03)	0.86(0.57, 1.29)
		Negative pre-vaccination	116	100%	426.65	365.05, 498.63	0.00(-3.21, 3.15)	0.81(0.66, 0.99)
	HPV-18 (IU/mL)	Total	156	100%	318.30	276.64, 366.24	0.00(-2.40, 2.51)	0.78(0.64, 0.96)
		Positive pre-vaccination	22	100%	376.49	220.93, 641.58	0.00(-14.87, 22.81)	1.07(0.48, 2.35)
		Negative pre-vaccination	134	100%	309.64	268.86, 356.62	0.00(-2.79, 2.75)	0.75(0.61, 0.93)
	HEV(WU/mL)	Total	156	100%	10.51	9.43, 11.71	0.00(-2.40, 2.50)	0.94(0.81, 1.09)
		Positive pre-vaccination	3	100%	20.90	7.28, 59.99	0.00(-56.15, 39.03)	1.28(0.68, 2.42)
		Negative pre-vaccination	153	100%	10.37	9.30, 11.56	0.00(-2.45, 2.60)	0.94(0.81, 1.09)
Group B: HPV	HPV-16(IU/mL)	Total	149	100%	529.20	466.89, 599.81	–	–
		Positive pre-vaccination	31	100%	528.93	377.20, 741.69	–	–
		Negative pre-vaccination	118	100%	529.27	462.94, 605.10	–	–
	HPV-18(IU/mL)	Total	149	100%	407.54	350.50, 473.86	–	–
		Positive pre-vaccination	13	100%	352.59	201.07, 618.29	–	–
		Negative pre-vaccination	136	100%	413.22	352.70, 484.12	–	–
Group C: HEV	HEV(WU/mL)	Total	150	100%	11.18	10.11, 12.37	–	–
		Positive pre-vaccination	6	100%	16.26	11.16, 23.70	–	–
		Negative pre-vaccination	144	100%	11.01	9.93, 12.21	–	–

^a^ : Non-inferior (lower limit of the 95% CI for the difference in antibody seroconversion rates in HPV+HEV group minus HPV group was greater than -5%).

^b^ : Non-inferior (lower limit of the 95% CI for the ratio of GMC between the HPV+HEV group and the HPV group was not less than 0·5).

GMC, geometric mean concentration.

Overall, all vaccines administered during the trial period were well-tolerated, the majority of ARs across all groups were Grade 1, with few Grade 2 and 3 observed. (**Table 3**) The incidence of overall ARs was significantly higher at co-administration group than HPV-only administrations but lower than HEV-only group.

**Table 3:** Percentage of doses followed by solicited local and general symptoms during the 7-day post-vaccination period (SS cohort).

Type of adverse reactions	Group A HPV+HEV(n=474)	Group B HPV(n=467)	Group C HEV(n=477)	χ² ^a^	p-value ^a^	χ² ^b^	p-value ^b^
Local symptoms	103(21.7)	14(3.0)	106(22.2)	74.102	<0.001	0.011	0.916
Redness	2(0.4)	0(0.0)	5(1.0)	-	0.500^d^	-	0.451^d^
Swelling	4(0.8)	1(0.2)	13(2.7)	-	0.373^d^	3.782	0.052
Pain	46(9.7)	11(2.4)	40(8.4)	21.056	<0.001	0.355	0.551
Induration	32(6.8)	0(0.0)	21(4.4)	-	<0.001^d^	2.066	0.151
Pruritus	19(4.0)	2(0.4)	27(5.7)	12.227	<0.001	1.073	0.300
General symptoms	22(4.6)	12(2.6)	52(10.9)	2.335	0.126	12.126	<0.001
Fever	12(2.5)	12(2.6)	19(4.0)	0.000^e^	1.000	1.162	0.281
Cough	0(0.0)	0(0.0)	1(0.2)	-	1.000^d^	-	1.000^d^
Headache	4(0.8)	0(0.0)	8(1.7)	-	0.124^d^	0.741	0.390
Myalgia	1(0.2)	0(0.0)	9(1.9)	-	1.000^d^	-	0.021^d^
Fatigue	2(0.4)	0(0.0)	8(1.7)	-	0.500^d^	-	0.108^d^
Gastrointestinal ^c^	3(0.6)	0(0.0)	7(1.5)	-	0.249^d^	-	0.341^d^
Overall adverse reaction grade	125(26.4)	26(5.6)	155(32.5)	74.037	<0.001	4.002	0.045
1	114(24.1)	26(5.6)	137(28.7)	62.006	<0.001	2.435	0.119
2	9(1.9)	0(0.0)	16(3.4)	-	0.004^d^	1.440	0.230
≥3	2(0.4)	0(0.0)	2(0.4)	-	0.500^d^	-	1.000^d^
Serious adverse reactions	0(0.0)	0(0.0)	0(0.0)	-	1.000^d^	-	1.000^d^

^a^ : Group A compared with Group B.

^b^ : Group A compared with Group C.

^c^ : Gastrointestinal symptoms included nausea, vomiting, diarrhea and/or abdominal pain.

^d^ : Fisher’s exact test.

^e^ : Chi-square (χ²) values are reported to three decimal places; values < 0·001 are presented as 0·000.

Group A demonstrated significantly higher occurrences of both grade 1 and grade 2 reactions relative to Group B. Redness, swelling, pain, induration and pruritus were reported to vary degrees, with some participants experiencing multiple concurrent local adverse reactions. Fever, headache, myalgia, fatigue and gastrointestinal symptoms such as nausea, vomiting, diarrhea and/or abdominal pain were the most frequently occurred general symptoms among participants in all groups. Group A showed a significantly higher overall incidence of local reactions, as well as higher rates for each individual reaction type except for redness and swelling, compared to Group B. No significant difference was observed in the incidence of general symptoms between Group A and Group B. Group C exhibited higher incidence rates than Group A for the majority of local and general symptoms, with overall general symptom rate and myalgia reaching statistical significance. Grade 3 solicited adverse reactions were observed in Group A (1 instance of fever, 1 instance of induration) and in Group C (2 instances of fever). No serious adverse reactions (SARs) were reported in any group during the study period.

The frequency of unsolicited adverse events and other safety outcomes is shown in **Table 4**. There were no deaths among any participants during the study period. No statistically significant differences were observed in either the number of participants reporting at least one event or the number of doses followed by an unsolicited adverse event, when comparing Group B with Group A or Group C with Group A.

**Table 4:** Frequency of unsolicited adverse events and other safety outcomes (SS cohort).

Safety outcomes	Group A HPV+HEV	Group B HPV	Group C HEV
Number of participants	159	160	161
Number of total doses	474	467	477
Number of participants reporting at least one event	21	16	24
Number of doses followed by any unsolicited adverse events (%)	26(5.49)	23(4.93)	27(5.66)
Number of doses followed by any grade 3 unsolicited adverse event (%)	2(0.42)	1(0.21)	1(0.21)
SAEs (%)	0(0.00)	1(0.21)	1(0.21)
Pregnancies (%)	1(0.63)	0(0.00)	0(0.00)

SAEs, serious adverse events.

Two serious adverse events (SAEs) were reported during the trial period. (See Appendix 3 and 4 of **Supplementary Materials**) One case of hyperinsulinemia was confirmed in Group B (Month 6), and one case of chronic nephritis occurred in Group C (Month 4). Both patients received prompt medical intervention and achieved favorable recovery outcomes. The participant in Group B completed the full trial protocol, while the participant in Group C withdrew from the study. According to the Chinese NMPA guideline (28, 29) and the WHO AEFI Causality Assessment tool (AEFICA), the two SAEs in our trial were both deemed unrelated to vaccination. Therefore, no vaccine-related SAEs occurred during the study period.

One pregnancy was reported in a 22-year-old girl in the HPV+HEV group. The girl had a negative urine pregnancy test at the baseline visit, and the pregnancy was identified at Month 4. She withdrew from the study and gave birth to a healthy boy after 9 months of pregnancy. Although no structured infant follow−up was mandated by the protocol, postnatal telephone follow−up confirmed that the infant’s health examinations at 28 days and 6 months were normal.

## 4 Discussion

This open-label, randomized, controlled phase 4 clinical trial demonstrated that co-administration of the HPV-16/18 and HEV vaccines was well tolerated and immunogenic in healthy females aged 18–25 years. This age group aligns with the range generally recommended for routine HPV vaccination (4, 30) and represents a key target population for hepatitis E vaccination (31). In this study, we demonstrated that immune responses to HPV-16/18 and HEV antigens following co-administration were at least as robust as those following administration of either vaccine alone in this population. Co-administration provides comparable levels of antibody protection to individual vaccination. The co-administered vaccination is well-tolerated, enhancing the feasibility of public health prevention strategies for both HPV and HEV.

As of August 2025, seven prophylactic HPV vaccines developed by five companies worldwide have received marketing authorization (5). Of these, six have been approved by China’s National Medical Products Administration (NMPA) for use in the country (32). Except for the domestically developed 9-valent HPV vaccine (derived from Escherichia coli), which was approved in May 2025, the other five HPV vaccines used in China have all received prequalification from the World Health Organization (WHO). HPV vaccines have now been included in China’s National Immunization Program (NIP) (33). In addition, numerous provinces and cities have integrated the vaccine into their local immunization programs (34), with the government procuring the vaccine and promoting its vaccination among adolescent females. Hepatitis E remains a major contributor to the global burden of viral hepatitis (35). Although the only available hepatitis E vaccine, HEV 239, has been proven safe and effective (18), its accessibility remains limited due to various challenges. Currently, this vaccine is licensed only in China, Pakistan, and India, and has not yet received WHO prequalification, limiting its widespread use in resource-limited, low-income countries (36). In this context, conducting studies on the immunogenicity and safety of co-administering the HPV-16/18 and HEV vaccines is essential to supplement post-marketing clinical data for both vaccines.

Following completion of the full immunization schedule, antibody levels against HPV-16/18 and HEV showed significant increases in all three groups. All participants who were seronegative for HPV-16/18 or HEV prior to vaccination seroconverted after completing the full vaccination series. Among participants who were seropositive at baseline, GMCs increased at least 4-fold post-vaccination compared to pre-vaccination levels. All participants in each group met the seroconversion criteria, indicating that both the co-administration and separate administration of the HPV-16/18 and HEV vaccines elicited similarly robust serological immune responses, with the combined regimen demonstrating non-inferiority to the individual vaccines. Both seroconversion rates and GMC ratios between the co-administration group and the groups receiving only the HPV-16/18 vaccine or only the HEV vaccine met the predefined non-inferiority criteria. Therefore, co-administration of the two vaccines demonstrates a favorable immunogenicity profile.

Although the incidence of ARs in the co-administration group was slightly higher than that in the HPV-only group, it was lower than that in the HEV-only group. ARs across all groups were predominantly Grade 1, with Grade 2 and 3 ARs being rare. No SARs were reported, and no vaccine-related SAEs occurred in either group during the study period, indicating that co-administration was well tolerated. The co-administration of HPV and HEV vaccines may have caused overactive immune responses. Research investigated the correlation between inflammation-related solicited adverse reactions and specific immune responses induced by Cecolin and HEV 239, which demonstrated that inflammation-related adverse reactions such as induration, redness, pain and fever may suggest a stronger immune response (37). From this perspective, a higher incidence of adverse reactions may indicate a stronger antibody response, suggesting that co-administration could potentially enhance the body’s immune response. While aluminum adjuvants are generally considered safe, higher cumulative doses have been associated with an increased likelihood of injection-site reactions in some studies (38, 39). Thus, the higher total aluminum load (485 μg) in the co-administration group might partially explain why the combined vaccination group had a significantly higher incidence of local adverse reactions compared to the individual vaccination group, but other factors cannot be ruled out. Additionally, most adverse reactions in the combined vaccination group were low-grade, with mild symptoms and rapid recovery. Overall, the safety profile of the combined vaccination group was well-tolerated.

To our knowledge, this is the first study to demonstrate the immunogenicity and safety of concomitant HPV and HEV vaccination. Given the similar vaccination schedules for HPV and HEV (both requiring the second and third doses at month 1 and 6), promoting combined HPV and HEV vaccination—while ensuring immunogenic efficacy and safety—can reduce the number of visits to healthcare facilities for vaccination, save time costs and enhance vaccination willingness. For women of reproductive age, completing effective vaccination against both HPV and HEV prior to pregnancy can significantly reduce the risk of developing cervical cancer and adverse pregnancy out-comes. Simultaneous administration increases the timeliness of vaccination in the tar-get population and improves overall vaccination coverage rates. Furthermore, the study had an adequate sample size, implemented strict participant screening, and achieved high completion rates, decreasing the potential for bias.

In this study, a non−negligible proportion of participants were seropositive for HPV−16 (21–26%) and HPV−18 (9–14%) at baseline. These baseline−positive individuals all exhibited ≥4−fold rises in antibody titers after full vaccination, indicating a strong anamnestic (boosting) response. However, pre−existing immunity could influence the GMC comparisons between groups if the proportion of baseline−positive subjects differs between the co−administration and HPV−alone groups. In our trial, the baseline seropositivity rates were well balanced between Group A and Group B (HPV−16: 26.4% vs. 21.3%; HPV−18: 13.8% vs. 8.8%; both p>0.05), minimizing the risk of bias. Moreover, the non−inferiority analysis for GMC ratios was also performed in the baseline−seronegative subset, which confirmed consistent results (e.g., HPV−16 GMC ratio 0.82 (95%CI: 0.68–0.98) in the full PPS vs. 0.81 (95%CI: 0.66–0.99) in the PPS subset; see **Supplementary Appendix 7**). Therefore, pre−existing immunity did not materially affect the non−inferiority conclusions.

The observed 100% seroconversion rates (for both HPV and HEV) are consistent with previous phase 3 trials of the same vaccines (7, 21). However, the lower limits of detection (LLOD) for HPV−16 (3.0 IU/mL) and HPV−18 (2.1 IU/mL) are relatively low, which may facilitate achieving 100% seroconversion. Even when applying a higher threshold (e.g., 10 IU/mL), the seroconversion rates remained > 99% (**Supplementary Appendix 8**), confirming the robust immunogenicity of both regimens.

Our study population consisted of healthy females aged 18–25 years, among whom natural HPV infection is common. The baseline seropositivity in this age group may be higher than that in younger adolescents (9–14 years) targeted by most HPV vaccination programs. Thus, the absolute GMC levels observed here might overestimate the response in a largely HPV−naïve population. Nevertheless, the non−inferiority comparison between co−administration and single vaccination is likely to be generalizable, as the relative effect (ratio) is less affected by baseline serostatus when groups are balanced.

This study has several unavoidable limitations. First, the HEV−alone group unexpectedly reported more adverse reactions than the co−administration group, a post−hoc observation that requires further mechanistic investigation. Second, although a higher aluminum load may be associated with an increased incidence of adverse reactions, the observation that systemic adverse reactions were significantly higher in the HEV alone group than in the combined vaccination group cannot be fully explained by the difference in aluminum load alone. Other potential mechanisms may be involved (40, 41), and further investigation is warranted to elucidate the underlying reasons for this finding. Third, we did not collect height or weight data, precluding BMI-based subgroup analyses; however, the study population was homogeneous in age and health status. Fourth, we did not record handedness or balance injection sides, which could theoretically affect local pain reporting, but this is unlikely to bias overall safety comparisons. Finally, the follow−up period in the current study was limited to one month after vaccination, and therefore long−term protective effects of the combined vaccination could not be assessed. In our subsequent research, we plan to extend the observation period to evaluate the durability of protection following co−administration.

## 5 Conclusions

Co-administration of the HPV and HEV vaccines induced high seroconversion rates and met non-inferiority criteria compared to administration of each vaccine alone, indicating that both simultaneous and separate administration can achieve robust and comparable immune responses. All reported ARs were predominantly Grade 1, and Grade 2 and 3 ARs were rare. No vaccine-related SAEs occurred in either group during the study period, indicating that co-administration was well tolerated. These findings suggest that co-administration of HPV and HEV vaccines in women of childbearing age is not only convenient and conducive to improved compliance, but also demonstrates favorable immunogenicity and safety.

## Data Availability

The raw data supporting the conclusions of this article will be made available by the authors, without undue reservation.

## Group member of RIDPHE Group

Innovation group on intelligent response to infectious diseases and public health emergencies. Shigui Yang, Xudong Zhou, Peige Song, Ning Zhang, Hao Lei, Junfang Xu, Jianbing Wang.
