# Supplementary material for: Immunogenicity and safety of co-administered Escherichia coli-produced bivalent HPV-16/18 vaccine and hepatitis E vaccine
Source: Front Immunol. 2026 May 29;17:1837539. doi: 10.3389/fimmu.2026.1837539 (PMC13260377; doi:10.3389/fimmu.2026.1837539)
Supplement: Supplementary file 1 [file SupplementaryFile1.docx]

Supplementary Material

Contents

[Appendix 1. Inclusion and exclusion criteria 1](#_Toc225186283)

[Inclusion criteria 1](#_Toc225186284)

[Exclusion Criteria 1](#_Toc225186285)

[Appendix 2. Sample Size 3](#_Toc225186286)

[Appendix 3. Post‑hoc power analysis for GMC ratio non‑inferiority 5](#_Toc225186287)

[Appendix 4. List of severe adverse events 6](#_Toc225186288)

[Appendix 5. List of pregnancies 7](#_Toc225186288)

[Appendix 6. Immunogenicity results in the ITT dataset (sensitivity analysis) 8](#_Toc225186288)

[Appendix 7. Geometric Mean Ratios of HPV16‑IgG, HPV18‑IgG, and HEV‑IgG Antibodies, and Differences in Seroconversion Rates with 95% Confidence Intervals at Month 7 9](#_Toc225186288)

[Appendix 8. Seroconversion rates for HPV16‑IgG and HPV18‑IgG at month 7 using a threshold of 10 IU/mL for both antibodies 1](#_Toc225186288)0

# Appendix 1. Inclusion and exclusion criteria

# Inclusion criteria:

1. Female, aged between 18 and 25 years (including 18 and 25 years) on the day of enrollment.
2. Judged as healthy and eligible for vaccination by the investigators through a self- reported medical history and some physical examinations.
3. Willing to participate in this study and sign informed consent form.
4. Able to understand this study information and willing to comply with all study requirements.
5. Axillary temperature ≤37.0 °C.
6. Negative urine pregnancy test.

**Exclusion Criteria:**

1. Women who are pregnant or breastfeeding or who plan to get pregnant within the next seven months.
2. Use of any investigational product or non-registered product (drug or vaccine) within 30 days preceding the first dose of the study vaccine or plan to use during the study period.
3. Received immunosuppressed, immunoregulation therapy or corticosteroid systemic therapy for more than 14 days in the 6 months before entry, except local treatment.
4. Administration of any immunoglobulin or blood products within 3 months preceding the first dose of the study vaccine, or plan to use during the study period.
5. Administration of any inactivated vaccines within 14 days preceding the first dose of the study or attenuated live vaccines within 21 days preceding the first dose of the study.
6. Had a fever (axillary temperature over 38°C) within 3 days or acute illness requiring systemic antibiotics or antiviral treatment within 5 days before vaccination.
7. Plan to participate in another clinical study at the same time during the study.
8. Previous vaccination against HPV or HEV.
9. Immunodeficiency (such as HIV positive), primary disease of important organs, malignant tumor, or any immune disease (such as systemic lupus erythematosus, arthritis pauperum, splenectomy or functional asplenia or other disease which might affect immune response).
10. History of allergic disease or history of serious adverse events occurring after vaccination, i.e., allergy, urticaria, dyspnea, angioneurotic edema or abdominal pain.
11. Asthma, which has been unstable for the past two years and requires urgent treatment, hospitalization, oral or intravenous corticosteroid.
12. Complicated with serious medical diseases, such as hypertension, heart disease, diabetes, hyperthyroidism, etc.
13. Medical diagnosis of abnormal coagulation function (such as coagulation factor deficiency, coagulation disorders, platelet abnormalities) or coagulation disorders.
14. Epilepsy, excluding febrile epilepsy under 2 years of age, alcoholic epilepsy 3 years before abstinence or simple epilepsy that did not require treatment for the past 3 years.
15. Past or present mental illness due to a psychological condition that does not comply with the requirements of the study; mental illness that has not been well controlled in the past two years; mental illness requiring medication; and suicidal tendency in the past five years.
16. Other medical, psychological, social or occupational factors that, according to the investigators' judgment, might affect the individual's ability to obey the protocol or sign the informed consent.

# Appendix 2. Sample Size

**Sample Size for Safety Evaluation.**

According to the requirements of the Guideline for Vaccine Clinical Trials (1), approximately 300 subjects are needed per vaccine to evaluate common adverse reactions. The study recruited participants in a 1:1:1 ratio among the HPV and HEV co-administration group, the HPV vaccine group, and the HEV vaccine group, with at least 150 participants in each group.

**Sample Size for Immunogenicity Evaluation**

1. Sample Size for Single-Group Immunogenicity Evaluation in Control Groups

PASS software (version 11.0, NCSS, LLC, Kaysville, UT, USA) was used to estimate the sample size for single-group immunogenicity evaluation. Using a two-sided significance level $\text{α}$ = 0.05, the required sample size per group under different assumed seroconversion rates was estimated based on target half-widths of the 95% CI of 0.20, 0.15, and 0.10.

**Table 1. Sample size estimation for immunogenicity evaluation in single groups**

| Seroconversion rate | 95% CI Half-width | | |
| --- | --- | --- | --- |
|  | 0.20 | 0.15 | 0.10 |
| 99.5 | 70 | 121 | 246 |
| 99 | 44 | 74 | 158 |
| 95 | 29 | 47 | 94 |
| 90 | 19 | 27 | 44 |
| 85 | 18 | 25 | 39 |

Considering that immunogenicity evaluation is required for each control vaccine group, with a target 95% CI half-width of 0.15, and assuming seroconversion rates of 99% for the HPV vaccine and 99.5% for the HEV vaccine, the HPV vaccine group requires 74 subjects and the HEV vaccine group requires 121 subjects. Accounting for a 10% baseline seropositivity rate and a 5% dropout rate, the required sample sizes are 88 and 143 subjects, respectively.

2. Sample Size for Inter-Group Immunogenicity Evaluation

We assumed HPV16-IgG and HPV18-IgG seroconversion rates of 99.0% in Groups A and B, and a 99.5% HEV-IgG seroconversion rate in Groups A and C. These assumptions were based on previous studies showing 100.0% seroconversion rates (2) for HPV16-IgG and HPV18-IgG one month after completion of the three-dose bivalent HPV-16/18 vaccine (E. coli-produced), and a 99.86% (3) HEV-IgG seroconversion rate one month after the third dose among baseline seronegative individuals.

**Table 2. Sample size estimation for immunogenicity evaluation between groups**

| Statistical power(1-β) | HPV | | HEV | |
| --- | --- | --- | --- | --- |
|  | Group A | Group B | Group A | Group C |
| 0.99 | 255 | 255 | 186 | 186 |
| 0.95 | 198 | 198 | 151 | 151 |
| 0.90 | 170 | 170 | 133 | 133 |
| 0.85 | 152 | 152 | 122 | 122 |
| 0.80 | 139 | 139 | 114 | 114 |
| 0.75 | 128 | 128 | 107 | 107 |
| 0.70 | 119 | 119 | 91 | 91 |

Non-inferiority was defined as a lower limit of the confidence interval for the seroconversion rate difference ≥ -5%. Using a one-sided significance level of 0.025, sample sizes for Groups A and B (for HPV) and Groups A and C (for HEV) were calculated at 80% power. The required sample size per group was 139, which, after adjusting for a 10% baseline seropositivity rate and a 5% dropout rate, increased to approximately 160 participants per group.

Thus, the HPV and HEV co-administration group (Group A), the HPV vaccine group (Group B), and the HEV vaccine group (Group C) will each enroll about 160 participants, totaling approximately 480.

The final sample size was harmonized across groups to ensure balanced allocation and sufficient power for both safety and immunogenicity assessments.

**References**

1 Center for Drug Evaluation, National Medical Products Administration. Technical guideline for vaccine clinical trials [Internet]. Beijing: Center for Drug Evaluation, NMPA; 2025 [cited 2026 Mar 20]. Available from: https://www.cde.org.cn/zdyz/opinioninfopage?zdyzIdCODE=3e23108aa065ae187ffb353794f7b1d4

2 Zhao F-H, Wu T, Hu Y-M, Wei L-H, Li M-Q, Huang W-J, Chen W, Huang S-J, Pan Q-J, Zhang X, et al. Efficacy, safety, and immunogenicity of an Escherichia coli-produced Human Papillomavirus (16 and 18) L1 virus-like-particle vaccine: end-of-study analysis of a phase 3, double-blind, randomised, controlled trial. Lancet Infect Dis (2022) 22:1756–1768. doi: 10.1016/S1473-3099(22)00435-2

3 Zhu F-C, Zhang J, Zhang X-F, Zhou C, Wang Z-Z, Huang S-J, Wang H, Yang C-L, Jiang H-M, Cai J-P, et al. Efficacy and safety of a recombinant hepatitis E vaccine in healthy adults: a large-scale, randomised, double-blind placebo-controlled, phase 3 trial. Lancet (2010) 376:895–902. doi: 10.1016/S0140-6736(10)61030-6

**Appendix 3. Post‑hoc power analysis for GMC ratio non‑inferiority**

The non‑inferiority margin for the GMC ratio (co‑admin / alone) was 0.5. Power was calculated for a one‑sided test at α=0.025 assuming a true ratio of 1.0 (equivalence). The standard deviation of log‑transformed GMCs (σ) was derived from the observed arithmetic mean (M) and standard deviation (SD) in the per‑protocol set using σ = √[ln(1+(SD/M)²)]. Results are summarized in the table below.

| Antigen | Group A (HPV+HEV) | Group B/C (alone) | σ (pooled) | Power |
| --- | --- | --- | --- | --- |
| HPV‑16 | n=156, GM=433.16 | n=149, GM=529.20 | 1.0 | >99.9% |
| HPV‑18 | n=156, GM=318.30 | n=149, GM=407.54 | 1.2 | 99.9% |
| HEV | n=156, GM=10.51 | n=150, GM=11.18 | 0.55 | >99.9% |

Even with σ as high as 1.4 (HPV‑18 worst case), power remains 99.0%. Therefore, the trial was adequately powered for the GMC‑based non‑inferiority endpoint.

# Appendix 4. List of severe adverse events

| **Group** | **Disease** | **Time of vaccination** | **Start time** | **End time** | **Outcome** | **Correlation with vaccination** |
| --- | --- | --- | --- | --- | --- | --- |
| Group B | Chronic nephritis IgA nephropathy | 16 January, 2022 | 4 February, 2022 | 10 February, 2022 | Improvement | NO |
| Group C | hyperinsulinemia | 3 November, 2021 | 5 April, 2022 | 12 April, 2022 | Improvement | NO |

# Appendix 5. List of pregnancies

| **Group** | Group A |
| --- | --- |
| **Time of vaccination** | 16 January, 2022 |
| **Diagnosis of Pregnancy Date** | 18 February, 2022 |
| **Last Menstrual Period Date** | 7 January, 2022 |
| **Pregnancy Outcome** | Completed delivery |
| **Date of Birth** | 20 October, 2022 |
| **Newborn Gender** | Male |
| **Mode of Delivery** | Vaginal delivery |
| **Newborn Birth Weight (g)** | 3720 g |
| **Gestational Age at Birth** | 40 weeks |
| **Were there any abnormalities or complications in the newborn of this pregnancy?** | NO |
| **Time of vaccination** | 16 January, 2022 |

# Appendix 6. Immunogenicity results in the ITT dataset (sensitivity analysis)

| Antigen | Group | N (seronegative at baseline) | Seroconversion rate % (95% CI) | GMC (95% CI) | GMC ratio (Co‑admin / Alone) (95% CI) | Non‑inferiority met? |
| --- | --- | --- | --- | --- | --- | --- |
| HPV-16 | A（HPV+HEV） | 156 | 100.00 (97.66, 100.00) | 433.16 (379.70,494.15) | 0.82 (0.68, 0.98) | Yes |
| HPV-16 | B（HPV） | 149 | 100.00 (97.55, 100.00) | 529.20 (466.89, 599.81) |  |  |
| HPV-18 | A（HPV+HEV） | 156 | 100.00 (97.66, 100.00) | 318.30 (276.64, 366.24) | 0.78 (0.64, 0.96) | Yes |
| HPV-18 | B（HPV） | 149 | 100.00 (97.55, 100.00) | 407.54 (350.50, 473.86) |  |  |
| HEV | A（HPV+HEV） | 156 | 100.00 (97.66, 100.00) | 10.51 (9.43, 11.71) | 0.97 (0.83, 1.12) | Yes |
| HEV | C（HEV） | 157 | 100.00 (97.68, 100.00) | 10.86 (9.78, 12.06) |  |  |

# Appendix 7. Geometric Mean Ratios of HPV16‑IgG, HPV18‑IgG, and HEV‑IgG Antibodies, and Differences in Seroconversion Rates with 95% Confidence Intervals at Month 7

| Antibody | Group | GMC ratio (Co‑admin / Alone) (95% CI) | Difference in seroconversion rates (Co‑admin minus Alone) (95% CI) |
| --- | --- | --- | --- |
| **ITT** |  |  |  |
| HPV16-IgG | A（HPV+HEV）& B (HPV) | 0.82 (0.68, 0.98) | 0.00 ( -2.40, 2.51) |
| HPV18-IgG | A（HPV+HEV）& B (HPV) | 0.78 (0.64, 0.96) | 0.00 ( -2.40, 2.51) |
| HEV-IgG | A（HPV+HEV）& C (HEV) | 0.97 (0.83, 1.12) | 0.00 ( -2.40, 2.39) |
| **PPS** |  |  |  |
| HPV16-IgG | A（HPV+HEV）& B (HPV) | 0.82 (0.68, 0.98) | 0.00 ( -2.40, 2.51) |
| HPV18-IgG | A（HPV+HEV）& B (HPV) | 0.78 (0.64, 0.96) | 0.00 ( -2.40, 2.51) |
| HEV-IgG | A（HPV+HEV）& C (HEV) | 0.94 (0.81, 1.09) | 0.00 ( -2.40, 2.50) |
| **PPS‑Seropositive** |  |  |  |
| HPV16-IgG | A（HPV+HEV）& B (HPV) | 0.86 (0.57, 1.29) | 0.00 ( -8.76, 11.03) |
| HPV18-IgG | A（HPV+HEV）& B (HPV) | 1.07 (0.48, 2.35) | 0.00 (-14.87, 22.81) |
| HEV-IgG | A（HPV+HEV）& C (HEV) | 1.28 (0.68, 2.42) | 0.00 (-56.15, 39.03) |
| **PPS-I（PPS-Seronegative）** | | | |
| HPV16-IgG | A（HPV+HEV）& B (HPV) | 0.81 (0.66, 0.99) | 0.00 ( -3.21, 3.15) |
| HPV18-IgG | A（HPV+HEV）& B (HPV) | 0.75 (0.61, 0.93) | 0.00 ( -2.79, 2.75) |
| HEV-IgG | A（HPV+HEV）& C (HEV) | 0.94 (0.81, 1.09) | 0.00 ( -2.45, 2.60) |

**Appendix 8. Seroconversion rates for HPV16‑IgG and HPV18‑IgG at month 7 using a threshold of 10 IU/mL for both antibodies**

|  | **Group A: HPV+HEV** | **Group B: HPV** |
| --- | --- | --- |
| **ITT** |  |  |
| HPV16-IgG |  |  |
| Seroconversion (%) | 156(100.0) | 148(99.33) |
| 95% CI | 97.66, 100.00 | 95.76, 99.97 |
| HPV18-IgG |  |  |
| Seroconversion (%) | 156(100.0) | 148(99.33) |
| 95% CI | 97.66, 100.00 | 95.76, 99.97 |
| **PPS** |  |  |
| HPV16-IgG |  |  |
| Seroconversion (%) | 156(100.0) | 148(99.33) |
| 95% CI | 97.66,100.00 | 95.76, 99.97 |
| HPV18-IgG |  |  |
| Seroconversion (%) | 156(100.0) | 148(99.33) |
| 95% CI | 97.66,100.00 | 95.76, 99.97 |
| **PPS‑Seropositive** |  |  |
| HPV16-IgG |  |  |
| Seroconversion (%) | 40 (100.00) | 30(96.77) |
| 95% CI | 89.09, 100.00 | 81.48, 99.83 |
| HPV18-IgG |  |  |
| Seroconversion (%) | 22 (100.00) | 12(92.31) |
| 95% CI | 81.50, 100.00 | 62.09, 99.60 |
| **PPS-I（PPS-Seronegative）** | |  |
| HPV16-IgG |  |  |
| Seroconversion (%) | 116 (100.00) | 118 (100.00) |
| 95% CI | 96.87, 100.00 | 96.92, 100.00 |
| HPV18-IgG |  |  |
| Seroconversion (%) | 134 (100.00) | 136 (100.00) |
| 95% CI | 97.28, 100.00 | 97.32, 100.00 |

*Baseline seropositivity was defined using the original assay thresholds (HPV‑16: ≥3.0 IU/mL; HPV‑18: ≥2.1 IU/mL). Seroconversion at month 7 was defined as antibody concentration ≥10 IU/mL, regardless of baseline status.
